# Supplementary material for: Performance of different automatic photographic identification software for larvae and adults of the European fire salamander
Source: PLoS One. 2024 Apr 4;19(4):e0298285. doi: 10.1371/journal.pone.0298285 (PMC10994360; doi:10.1371/journal.pone.0298285)

PLOS ONE

Performance of different automatic photographic identification software for larvae and adults of the European fire salamander

--Manuscript Draft--

**Manuscript Number:**	PONE-D-23-31936
**Article Type:**	Research Article
**Full Title:**	Performance of different automatic photographic identification software for larvae and adults of the European fire salamander
**Short Title:**	Photographic identification software for the European fire salamander
**Corresponding Author:**	Laura Schulte<br>Bielefeld University: Universitat Bielefeld<br>Bielefeld, GERMANY
**Keywords:**	Capture-recapture; photographic identification software; individual identification; <i>Salamandra salamandra<i/>
**Abstract:**	For many species, population sizes are unknown despite their importance for conservation. For population size estimation, capture-mark-recapture (CMR) studies are often used, which include the necessity to identify each individual, mostly through individual markings or genetic characters. Invasive marking techniques, however, can negatively affect the individual fitness. An alternative are non-invasive techniques, such as the use of photos for individual identification, at least for species with stable individual-specific colour patterns or other such markings. For the individual identification of photos, a variety of different software, with different requirements, is available. The European fire salamander (<i>Salamandra salamandra</i>) is a species in which individuals, both at the larval stage and as adults, have individual specific patterns that allow for individual identification. In this study, we compared the performance of five different computer-based software for the use of photographic identification for the European fire salamander: Amphibian & Reptile Wildbook (ARW), AmphIdent, I3S pattern+, ManderMatcher and Wild-ID. While adults can be identified by all five software, European fire salamander larvae can only be identified by two of the five (ARW and Wild-ID). We used one dataset of European fire salamander larvae pictures taken in the laboratory and tested this dataset in two of the five software (ARW and Wild-ID). We used another dataset of European fire salamander adult pictures taken in the field and tested this using all five software. We compared the requirements of all software on the pictures used and calculated the False Rejection Rate (FRR) and the Recognition Rate (RR). For the larval dataset (421 pictures) we found that the ARW and Wild-ID performed equally well for individual identification (99.6% and 100% Recognition Rate, respectively). For the adult dataset (377 pictures), we found the best False Rejection Rate in ManderMatcher and the highest Recognition Rate in the ARW. Additionally, the ARW is the only program that requires no image pre-processing. In times of amphibian declines, non-invasive photo identification software allowing capture-mark-recapture studies help to gain knowledge on population sizes, distribution, movement and demography of a population and can thus help to support species conservation.
**Order of Authors:**	Laura Schulte
	Charlotte Faul
	Pia Oswald, Dr
	Kathleen Preißler, Dr
	Sebastian Steinfartz, Prof. Dr.
	Michael Veith, Prof. Dr.
	Barbara Caspers, Prof. Dr.
**Additional Information:**	
**Question**	**Response**

Powered by Editorial Manager® and ProduXion Manager® from Aries Systems Corporation

**Financial Disclosure**<br><br>Enter a financial disclosure statement that describes the sources of funding for the work included in this submission. Review the submission guidelines for detailed requirements. View published research articles from *PLOS ONE* for specific examples.<br><br>This statement is required for submission and **will appear in the published article** if the submission is accepted. Please make sure it is accurate.<br><br>**Funded studies**<br>Enter a statement with the following details:<br>• Initials of the authors who received each award<br>• Grant numbers awarded to each author<br>• The full name of each funder<br>• URL of each funder website<br>• Did the sponsors or funders play any role in the study design, data collection and analysis, decision to publish, or preparation of the manuscript?<br><br>Did you receive funding for this work?	Yes
Please add funding details.<br>as follow-up to "**Financial Disclosure**<br><br>Enter a financial disclosure statement that describes the sources of funding for the work included in this submission. Review the submission guidelines for detailed requirements. View published research articles from *PLOS ONE* for specific examples.<br><br>This statement is required for submission and **will appear in the published article** if the submission is accepted. Please make sure it is accurate.	This research was conducted as part of the CRC TRR 212 (NC³) – Project number 316099922 and 396777092 funded by the German Research Foundation (DFG).

**Funded studies**<br>Enter a statement with the following details:<br>• Initials of the authors who received each award<br>• Grant numbers awarded to each author<br>• The full name of each funder<br>• URL of each funder website<br>• Did the sponsors or funders play any role in the study design, data collection and analysis, decision to publish, or preparation of the manuscript?<br><br>Did you receive funding for this work?"	
Please select the country of your main research funder (please select carefully as in some cases this is used in fee calculation).<br>as follow-up to "**Financial Disclosure**<br><br>Enter a financial disclosure statement that describes the sources of funding for the work included in this submission. Review the submission guidelines for detailed requirements. View published research articles from *PLOS ONE* for specific examples.<br><br>This statement is required for submission and **will appear in the published article** if the submission is accepted. Please make sure it is accurate.<br><br>**Funded studies**<br>Enter a statement with the following details:<br>• Initials of the authors who received each award<br>• Grant numbers awarded to each author<br>• The full name of each funder<br>• URL of each funder website<br>• Did the sponsors or funders play any role in the study design, data collection and analysis, decision to publish, or preparation of the manuscript?<br><br>Did you receive funding for this work?"	GERMANY - DE
**Competing Interests**	The department of Behavioural Ecology from Bielefeld University placed the order for

Powered by Editorial Manager® and ProduXion Manager® from Aries Systems Corporation

Use the instructions below to enter a competing interest statement for this submission. On behalf of all authors, disclose any competing interests that could be perceived to bias this work—acknowledging all financial support and any other relevant financial or non-financial competing interests.<br><br>This statement is required for submission and **will appear in the published article** if the submission is accepted. Please make sure it is accurate and that any funding sources listed in your Funding Information later in the submission form are also declared in your Financial Disclosure statement.<br><br>View published research articles from *PLOS ONE* for specific examples.<br><br>**NO authors have competing interests**<br><br>Enter: *The authors have declared that no competing interests exist.*<br><br>**Authors with competing interests**<br><br>Enter competing interest details beginning with this statement:<br><br>*I have read the journal's policy and the authors of this manuscript have the following competing interests: [insert competing interests here]*<br><br>* typeset	the Amphibian & Reptile Wildbook software together with NC3 (Collaborative research centre "A Novel Synthesis of Individualisation across Behaviour, Ecology and Evolution: Niche Choice, Niche Conformance, Niche Construction (NC3)" founded by the DZG) and Heike Pröhl and Mirjam Nadjafzadeh, who received funding from the Deutsche Gesellschaft für Herpetologie und Terrarienkunde (DGHT). They are not receiving any kind of payment or other benefits in relation with the Amphibian & Reptile Wildbook.
**Ethics Statement**<br><br>Enter an ethics statement for this submission. This statement is required if the study involved:<br><br>• Human participants<br>• Human specimens or tissue<br>• Vertebrate animals or cephalopods<br>• Vertebrate embryos or tissues	N/A

• Field research<br><br>Write "N/A" if the submission does not require an ethics statement.<br><br>General guidance is provided below. Consult the submission guidelines for detailed instructions. **Make sure that all information entered here is included in the Methods section of the manuscript.**	

Powered by Editorial Manager® and ProduXion Manager® from Aries Systems Corporation

**Format for specific study types**<br><br>**Human Subject Research (involving human participants and/or tissue)**<br>• Give the name of the institutional review board or ethics committee that approved the study<br>• Include the approval number and/or a statement indicating approval of this research<br>• Indicate the form of consent obtained (written/oral) or the reason that consent was not obtained (e.g. the data were analyzed anonymously)<br><br>**Animal Research (involving vertebrate animals, embryos or tissues)**<br>• Provide the name of the Institutional Animal Care and Use Committee (IACUC) or other relevant ethics board that reviewed the study protocol, and indicate whether they approved this research or granted a formal waiver of ethical approval<br>• Include an approval number if one was obtained<br>• If the study involved *non-human primates*, add *additional details* about animal welfare and steps taken to ameliorate suffering<br>• If anesthesia, euthanasia, or any kind of animal sacrifice is part of the study, include briefly which substances and/or methods were applied<br><br>**Field Research**<br><br>Include the following details if this study involves the collection of plant, animal, or other materials from a natural setting:<br>• Field permit number<br>• Name of the institution or relevant body that granted permission	
**Data Availability**<br><br>Authors are required to make all data underlying the findings described fully available, without restriction, and from the time of publication. PLOS allows rare exceptions to address legal and ethical concerns. See the PLOS Data Policy and FAQ for detailed information.	No - some restrictions will apply

Powered by Editorial Manager® and ProduXion Manager® from Aries Systems Corporation

A Data Availability Statement describing where the data can be found is required at submission. Your answers to this question constitute the Data Availability Statement and **will be published in the article**, if accepted.<br><br>**Important:** Stating 'data available on request from the author' is not sufficient. If your data are only available upon request, select 'No' for the first question and explain your exceptional situation in the text box.<br><br>Do the authors confirm that all data underlying the findings described in their manuscript are fully available without restriction?	
**Describe where the data may be found in full sentences. If you are copying our sample text, replace any instances of XXX with the appropriate details.**<br><br>• If the data are **held or will be held in a public repository**, include URLs, accession numbers or DOIs. If this information will only be available after acceptance, indicate this by ticking the box below. For example: *All XXX files are available from the XXX database (accession number(s) XXX, XXX.).*<br>• If the data are all contained **within the manuscript and/or Supporting Information files**, enter the following: *All relevant data are within the manuscript and its Supporting Information files.*<br>• If neither of these applies but you are able to provide **details of access elsewhere**, with or without limitations, please do so. For example:<br><br>*Data cannot be shared publicly because of [XXX]. Data are available from the XXX Institutional Data Access / Ethics Committee (contact via XXX) for researchers who meet the criteria for access to confidential data.*<br><br>*The data underlying the results presented in the study are available from (include the name of the third party*	Picture datasets used are available from the respective co-authors upon request because the picture datasets are very large and because of copyright reasons of the pictures.

and contact information or URL).<br>• This text is appropriate if the data are owned by a third party and authors do not have permission to share the data.<br><br>* typeset	
Additional data availability information:	

Manuscript

Click here to access/download;Manuscript;Manuscript_Schulte.docx

Performance of different automatic photographic identification software for larvae and adults of the European fire salamander

Laura Schulte[1]*, Charlotte Faul[2], Pia Oswald[1], Kathleen Preißler[3], Sebastian Steinfartz[3], Michael Veith[2t] & Barbara A. Caspers[14t]

[1] Department of Behavioural Ecology, Bielefeld University, Konsequenz 45, 33615 Bielefeld, Germany

[2] Biogeography, Trier University, Universitätsring 15, 54296 Trier, Germany

[3] Molecular Evolution and Systematics of Animals, Leipzig University, Talstraße 33, 04103 Leipzig

[4] JICE, Joint Institute for Individualisation in a Changing Environment, University of Münster and Bielefeld University, Germany

* Corresponding author: Laura Schulte, Schulte.Lra@gmail.com

[t] shared the last authorship

Keywords

Capture-recapture, photographic identification software, individual identification, *Salamandra salamandra*

Abstract

For many species, population sizes are unknown despite their importance for conservation. For population size estimation, capture-mark-recapture (CMR) studies are often used, which include the necessity to identify each individual, mostly through individual markings or genetic characters. Invasive marking techniques, however, can negatively affect the individual fitness. An alternative are non-invasive techniques, such as the use of photos for individual identification, at least for species with stable individual-specific colour patterns or other such markings. For the individual identification of photos, a variety of different software, with different requirements, is available. The European fire salamander (*Salamandra salamandra*) is a species in which individuals, both at the larval stage and as adults, have individual specific patterns that allow for individual identification. In this study, we compared the performance of five different computer-based software for the use of photographic identification for the

European fire salamander: Amphibian & Reptile Wildbook (ARW), AmphIdent, I3S pattern+, ManderMatcher and Wild-ID. While adults can be identified by all five software, European fire salamander larvae can only be identified by two of the five (ARW and Wild-ID). We used one dataset of European fire salamander larvae pictures taken in the laboratory and tested this dataset in two of the five software (ARW and Wild-ID). We used another dataset of European fire salamander adult pictures taken in the field and tested this using all five software. We compared the requirements of all software on the pictures used and calculated the False Rejection Rate (FRR) and the Recognition Rate (RR). For the larval dataset (421 pictures) we found that the ARW and Wild-ID performed equally well for individual identification (99.6% and 100% Recognition Rate, respectively). For the adult dataset (377 pictures), we found the best False Rejection Rate in ManderMatcher and the highest Recognition Rate in the ARW. Additionally, the ARW is the only program that requires no image pre-processing. In times of amphibian declines, non-invasive photo identification software allowing capture-mark-recapture studies help to gain knowledge on population sizes, distribution, movement and demography of a population and can thus help to support species conservation.

Introduction

Worldwide, 41% of the amphibian species suffer from population declines, and the conservation status of an additional 23% is not even known due to deficient data (IUCN, 2022; Stuart et al., 2004). Thus, knowledge of the ecology of these species, including population size, is crucial for implementing conservation measures (Sacchi et al., 2010). Capture-mark-recapture (CMR) studies are often used to estimate abundance of animals, to track life-histories and movements of individuals and to monitor the dynamic of communities and are a useful tool to estimate population sizes (Bolger et al., 2012; Drew et al., 2012; Gould and Kendall, 2013; Schulte et al., 2007).

Reliable population size estimates based on CMR studies require the recognition of an individual and thus appropriate marking techniques, which depend on the size, life stage and the studied species itself. For instance, RFID tags can be used for insects, radio transmitters or rings for birds, passive integrated transponder (PIT) tags in crustaceans and tattoos for mammals (Donnelly et al., 1994; Drew et al., 2012; Seber and Schofield, 2019). Furthermore, such markings should be permanent or at least last for the duration of the experiment (Aevarsson et al., 2022; Donnelly et al., 1994). However, often high costs are included for invasive tags

(Seber and Schofield, 2019), which is an issue in both ecological research and conservation projects.

Due to their permeable and sensitive skin, amphibians are difficult to tag (Heemeyer et al., 2007, but see e.g. Schulte et al., 2007 for a study on PIT tags inserted in the skin). Particularly marking amphibian larvae is challenging and there are only few techniques possible, such as fin clipping or staining (reviewed in Donnelly et al., 1994). Other invasive marking techniques for amphibians, such as toe clipping, do not only concern ethical issues (Perry et al., 2011) and increase handling time, they also increase stress for the organism (Roberts et al., 2021), may enhance infections, can reduce individual fitness and may ultimately affect survival rates (Bloch and Irschick, 2005; Donnelly et al., 1994), thus confounding population size estimates. Alternatively, PIT tags, inserted under the skin, have been proven to have no negative effect on adult European fire salamanders (*Salamandra salamandra*), making them a useful tool for marking species with a less distinct or no colour pattern, such as the alpine salamander (*S. atra*) (Schulte et al., 2007). In contrast, the use of Visible Implant (VI) Alpha tags in larvae of the European fire salamander negatively influenced their fitness (Wagner et al., 2020).

Photographic identification is a widely acknowledged non-invasive marking method. It provides an ideal way to recognize individuals without the potential negative effects of invasive markings (Cruickshank and Schmidt, 2017; Knox et al., 2013) and was already successfully used in a variety of amphibian species (Sannolo et al., 2016). Moreover, natural marks and patterns of animals usually cannot get lost, moulted or shed (Marshall and Pierce, 2012; Sannolo et al., 2016), although some animals have patterns that change over time with age such as in Grass Snakes (*Natrix helvetica*) (Baker and Allain, 2020). To enhance photo-identification, the use of photo recognition software has been proven useful. It is faster than the inspection by eye, less error-prone and also less prone to observer bias (Bolger et al., 2012; Cruickshank and Schmidt, 2017; Mettouris et al., 2016). It allows handling large datasets (Arzoumanian et al., 2005; Mettouris et al., 2016) and can be less expensive than other marking techniques (Bendik et al., 2013).

European fire salamanders show an individual-specific pattern, both during the larval stage as well as after metamorphosis, which allows individual identification (Eitam and Blaustein, 2002; Faul et al., 2022; Matthé et al., 2017; Speybroeck and Steenhoudt, 2017). The European fire salamander is widely distributed across Europe and inhabits preferably deciduous forests with access to water bodies for larval deposition (Thiesmeier, 2004). In Europe, the species is

listed as Least Concern (IUCN SSC Amphibian Specialist Group, 2022). However, in Germany the European fire salamander is now listed on the pre-warning list (Schlüpmann and Veith, 2020). The spread of the chytrid fungus *Batrachochytrium salamandrivorans* (*Bsal*) in Europe and particularly in Germany might further negatively affect European fire salamander populations (Lötters, 2020). Therefore, populations of the species are increasingly monitored (e.g., Oswald et al., 2023; Sandvoß et al., 2020; Wagner et al., 2020).

The performance of photographic identification has already been evaluated in several studies. These studies compared the between-observer variability when matching by eye vs. recognition software (Cruickshank and Schmidt, 2017), tested the performance of different matching algorithms (e.g. Matthé et al., 2017), or a completely different data source and matching algorithm (e.g. genetic data; Drechsler et al., 2015). Their findings suggest that the use of photographic matching software is better than matching by eye and that the algorithm used by a software strongly influences the matching results. The Amphibian and Reptile Wildbook (ARW) is a recently launched new pattern recognition software which is at the moment available for adults of the Yellow-bellied toad (*Bombina variegata*) and adult as well as larval pictures of the European fire salamander and the Near-eastern fire salamander (*S. infraimmaculata*). In contrast to other recognition software, it aims at a broad application in citizen science projects and constitutes an online option for the analysis of images of European fire salamanders that have not been pre-processed. The performance of the programme has not yet been tested in comparison to existing photo-identification software. We therefore use one adult and one larval European fire salamander dataset to compare software for individual pattern recognition. The adult dataset was tested on five software, whereas the larval dataset was tested on two software only due to software limitations and picture requirements. We used the following five software: I3S and Wild-ID, as they were already commonly used for CMR studies (e.g. Halloran et al., 2015; Rossi de Gasperis et al., 2017), and ARW, AmphIdent and ManderMatcher, which were especially developed for amphibian studies or studies on European fire salamanders (Goedbloed et al., 2017; Speybroeck and Steenhoudt, 2017). The aim of this study was to compare the different software by comparing the Recognition Rate (RR), False Rejection Rate (FRR) and the pre-processing requirements.

Methods

Compared software

Amphibian & Reptile Wildbook

ARW is a free and web-based software that was released in 2021 (https://amphibian-reptile.wildbook.org). It can be accessed online anywhere at any time. If the users upload their pictures publicly, it allows them to compare own captures with captures of other users that might have encountered the same individual (Blount et al., 2022). In this way, citizen scientists can contribute to research and conservation with their encounters of animals by enriching an existing database. Currently, ARW is validated for adults of Yellow-bellied toads, as well as larvae and adults of the European fire salamander and the Near eastern fire salamander. ARW does not require processing of the uploaded images (table 1), as the Hotspotter algorithm will automatically detect the position of the animal in the picture. It has recently been successfully used for a population size analysis of European fire salamander larvae in Germany (Oswald et al., 2023).

AmphIdent

AmphIdent is a commercial standard program for the identification of individuals of amphibian species. Different modules have been developed, such as for the great crested newt (*Triturus cristatus*), the European fire-bellied toad (*B. bombina*), the Yellow-bellied toad, the European fire salamander and the Marbled salamander (*Ambystoma opacum*). The software can be purchased at www.amphident.de. It requires the pre-processing of each picture as the body part to be compared needs to be chosen from the picture (e.g. the dorsal pattern of adult European fire salamanders). AmphIdent uses a pixel-based algorithm which was proven to be very robust with a large dataset in comparison to other recognition software (Drechsler et al., 2015; Matthé et al., 2017).

Interactive Individual Identification System (I3S)

I3S is a freely available software (https://reijns.com/i3s/) and was originally developed for sharks (Van Tienhoven et al., 2007). It now offers a suite of software solutions depending on the individual marking of the study species, e.g. spots for I3S classic. In each software, image processing is needed as the user needs to choose reference points in each image (Van Tienhoven et al., 2007). I3S uses the Scale Invariant Feature Transform (SIFT) algorithm which has been found to be very stable and repeatable regarding its performance (Sannolo et al., 2016).

It was successfully used for different species, such as two European lizard species (Sacchi et al., 2010), and I3S pattern works well for amphibians with large spots such as the Italian crested newt (*Triturus carnifex*) (Sannolo et al., 2016). For this study, I3S pattern+ was chosen due to suitability to recognise the striped pattern.

ManderMatcher

ManderMatcher is a free and pattern based tool (https://jeroenspeybroeck.shinyapps.io/mandermatcher/) that was successfully applied for *S. salamandra* but can also be used for other *Salamandra* species such as *S. algira, S. corsica* and *S. infraimmaculata* (Speybroeck and Steenhoudt, 2017). Image processing is required as every image needs to be coded after the salamanders body part, e.g. 11 characteristics need to be coded for the head (Speybroeck and Steenhoudt, 2017).

Wild-ID

Wild-ID is a free available software (https://faculty-directory.dartmouth.edu/douglas-thomas-bolger) that uses the SIFT operator. This operator detects characteristic image features that can then be compared over a large dataset (Bolger et al., 2012; Lowe, 2004). Wild-ID can be used for all species that show a distinctive colour pattern (Cruickshank and Schmidt, 2017). For better matching results, the software requires each picture to be cropped to the area of interest (Bolger et al., 2012). Wild-ID has been successfully used to study amphibians before, e.g., the Yellow-bellied toad and the Lake Oku clawed frog (*Xenopus longipes*) (Aevarsson et al., 2022; Cruickshank and Schmidt, 2017). In particular, even amphibian larvae can be identified, for example by comparing the pattern on the tail fin pattern of larvae of the European fire salamander (Faul et al., 2022).

Table 1: Image processing requirements and species availability for each software.

	Image processing required?	Available for which species?
Amphibian & Reptile Wildbook (ARW)	No	Yellow-bellied toad (*Bombina variegata*), European fire salamander (*Salamandra salamandra*), Near-eastern fire salamander (*S. infraimmaculata*)
AmphIdent	Yes	Great crested newt (*Triturus cristatus*), European fire-bellied toad (*B. bombina*), the Yellow-bellied toad, European fire salamander, the Marbled salamander (*Ambystoma opacum*)

I3S pattern+	Yes	Every species with individual pattern
ManderMatcher	Yes	Fire salamander species
Wild-ID	Yes	Every species with individual pattern

Used datasets

Larval dataset & mirrored larvae test

In the study of Faul et al. (2022), 40 European fire salamander larvae were captured in the Hunsrück Mountains (Germany) and kept under standardized conditions for up to ten weeks until the onset of metamorphosis. Starting with the second week, each larva was photographed under standardized conditions on a weekly basis (for further details see Faul et al., 2022). Hence, from week 2 on, every picture mimics a recapture event. We used the pictures from Faul et al. (2022) from the left body side (291 pictures, including 251 recaptures, table 2) to compare software performance. Like Faul et al. (2022), we included 130 additional pictures of wild caught larvae from the same creek to the dataset, as the performance of photo-matching software is known to decrease with an increasing dataset (Matthé et al., 2017). Due to the specific spots on the tail fin, only Wild-ID and ARW can process the pictures and match individuals among the compared software. For the use in Wild-ID, all pictures have been pre-processed by Faul et al. (2022) in a way that the part of the tail from the base of the hind leg to the end of the tail was cut out in a ratio 10:3.5. For the use in the ARW software, the pictures were not pre-processed.

For the mirrored larvae test, we have used a subset of the larval dataset. Faul et al. (2022) identified individual larvae by using only photos from the same body side. However, this can be time-consuming, especially in ~~in~~ the field, and cause additional stress for the larvae as handling time increases. Since larval tails are translucent and parts of the pattern of one side are always visible from the respective other side, we tested whether larvae could be reliably identified even when the photographed side of the larvae was not the same. We selected a random subset of 30 pictures from Faul et al. (2022) from the other body side (here the right one). We digitally mirrored these images (figure 1) and tested if the ARW and Wild-ID were able to find correct matches among the complete dataset of the images of the left body sides.

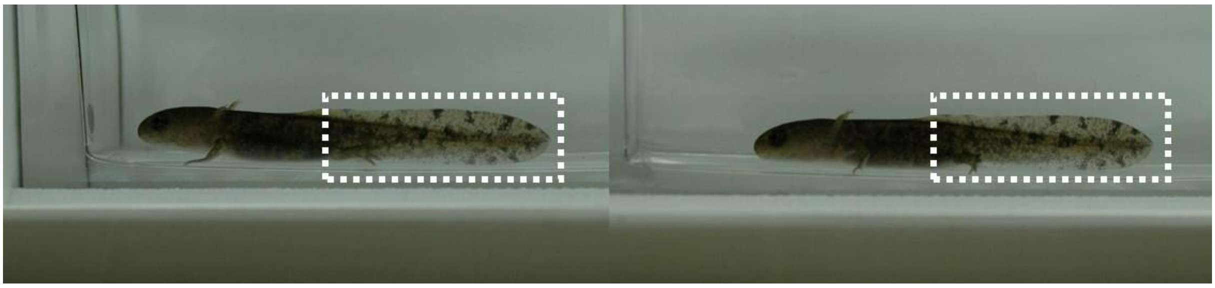

Figure 1: Two pictures of the same individual of a European fire salamander larva taken in week two of the experiment. Left: Picture taken from the left body side of the individual. Right: Picture taken from the right body side of the individual and then mirrored on the computer. White dashed boxes show the tail in which the upper and lower part are translucent whereas the centre is not translucent (pictures: Charlotte Faul).

Adult dataset

Between 2018 and 2020, 377 pictures of adult European fire salamanders were taken in the Kruppwald forest in the city of Essen (Germany). These are images of a population infected with *Bsal*. Images were taken from the dorsal side under non-standardized conditions in the field with varying backgrounds, such as forest floor, graph paper and sometimes even in the hand leading to an inhomogeneous dataset with different qualities of the pictures. The total dataset included 66 recaptures (17.5% recapture rate), identified via visual inspection in combination with the results of all software tested here. For visual inspection, two people manually compared all the pictures and assigned the recaptures by eye.

Recognition Rate (RR) & False Rejection Rate (FRR)

To quantify software performance, we calculated the Recognition Rate (%) and the False Rejection Rate (%) for each dataset and for each software separately. For the larval dataset in Wild-ID, we used the existing FRR from Faul et al. (2022). The FRR describes the probability of failing to recognise two pictures of the same individual within a given dataset (Bolger et al., 2012) and was calculated following Bolger et al. (2012) and Faul et al. (2022). We used it in three versions (Bolger et al., 2012; Faul et al., 2022): FRR1 refers to cases where a software was unable to place a true match in the first position; FRR10 refers to cases where no true matches were placed among the ten highest ranking images; FRR21 refers to cases where a software was unable to assign a correct image of the same individual to a test image within the 20 potential matches. Thus, a high FRR value indicates poor recognition, while a low FRR indicates

successful recognition of two pictures of the same individual. All software software require the observer to confirm or reject the proposed matches.

Table 2: Tested datasets and software used per dataset.

Dataset	Number of pictures per dataset	Software used
Larval dataset	421	ARW, Wild-ID
Mirrored larvae test	30	ARW, Wild-ID
Adult dataset	377	ARW, AmphIdent, I3S pattern+, ManderMatcher, Wild-ID

Results & Discussion

Larval dataset & mirrored larvae test

ARW and Wild-ID performed almost equally well when matching larval pictures, regarding both the RR and the FRR (Table 3). ARW was not able to match two pictures of the same amelanistic larva, while Wild-ID was able to match the pictures.

Table 3: Recognition Rate (RR, %) and False Rejection Rates FRR1, FFR10 and FRR21 (all in %) for the larval European fire salamander datasets.

Dataset	RR	FRR1	FRR10	FRR21
Larvae				
ARW	99.60	0.24	0.24	0.24
Wild-ID	100.00	3.52	0.00	0.00
Mirrored larvae test				
ARW	96.67	0.01	0.00	0.00
Wild-ID	100.00	0.24	0.00	0.00

The FFR showed some dissimilarity between Wild-ID and ARW. We found a higher FRR1 for Wild-ID compared to ARW (3.52% and 0%, respectively), while again the FFR10 and the FRR21 were almost identical for Wild-ID and ARW (table 3). All FRR values from both software can be considered very low and thus indicate a reliable image detection. Another study compared the tail fin venation of amphibian larvae by untrained citizen scientist by eye. Here, they demonstrated that the consensus of all participants on matches of *Litoria aurea* tadpoles varied

between 67% and 83% (Gould et al., 2023) and thus showing a lower Recognition Rate in comparison to the software indicating that the use of a software outcompetes by eye matching of (untrained) citizen scientists. Hence, both software reflect a reliable and beneficial tool in European fire salamander larvae research and conservation.

Furthermore, we tested the performance of ARW and Wild-ID by applying mirrored larval images to both software applications. In both cases, we found a very low FFR and a very high RR, the latter being 96.67% and 100%, respectively (table 3). This shows that both applications reliably match an image of one side of the body to an image of the other side of the body, which is important to have in mind when handling of larvae during fieldwork. Pictures of either body side can be taken in the field; this reduces stress to the individuals and time of field work. However, the time for subsequent processing of the images on the computer is increased.

Adult dataset

In our adult dataset, we found great differences among the software we tested. AmphIdent and I3S pattern+ performed comparably well, with the RR being higher for AmphIdent and the FRR1 and the FRR10 being lower for I3S pattern+ (table 4). Both software have also been used very successfully in other studies on amphibians and reptiles (e.g., Matthé et al., 2017; Sacchi et al., 2010; Sannolo et al., 2016). However, in a study on Great crested newts, Drechsler et al. (2015) found a low FRR of only 2% for AmphIdent. Nevertheless, Drechsler et al. (2015) also showed that pictures with poor quality (e.g., blurriness) could not be processed with AmphIdent and needed to be excluded from their study. While AmphIdent is available for only a few species, I3S is offering a suite of software solutions for species with different types of patterns. Goedbloed et al. (2017) demonstrated that AmphIdent produces much smaller FRR values for standardized (FRR = 0%) than for non-standardized (FRR = 35.2%) pictures, suggesting to always take standardized pictures with specific angles. Similarly, for I3S, Sacchi et al. (2010) found a high RR of 98%-99% in a lizard dataset; they had captured each lizard and photographed the ventral side of the body, thus also creating a highly standardized dataset. This can however be challenging during fieldwork if the animals are not captured as they often move or can be partially hidden by vegetation, which is hard to control. Thus, additional handling may be needed.

Table 4: Recognition Rate (RR, %) and False Rejection Rates FRR1, FFR10 and FRR21 (all in %) for the adult datasets; the combination of visual inspection and software results was used to define 100% of recaptures.

Dataset	RR	FRR1	FRR10	FRR21
ARW	77.27	10.88	6.90	3.98
AmphIdent	60.61	12.47	8.22	7.43
I3S pattern+	56.06	10.61	7.69	7.69
ManderMatcher	72.73	9.81	5.31	5.04
Wild-ID	37.88	15.12	12.47	10.88
Visual inspection	93.94	-	-	-
Visual & Software	100.00	-	-	-

While Wild-ID performed well for the larval dataset, performance for the adult dataset was much lower as indicated by the highest False Rejection Rates found in our study for FFR1, FFR10 and FRR21 (15.12%, 12.47% and 10.88%, respectively) and the lowest RR values with only 37.88%. In line with our results, Matthé et al. (2017) also found an RR value of only 30-49% when Wild-ID was used to analyse a European fire salamander dataset. In our study, we had trimmed all pictures prior to matching to increase the matching probability (Bolger et al., 2012). One explanation for this low performance could be that the adult images, unlike the larval images, were not taken under standardized conditions, and although the implemented SIFT operator should account for rotations and different views (Lowe, 2004), in most cases the animal's positions may have been too variable for successful matching. In previous studies, Wild-ID has been demonstrated to match images very successfully (e.g., Bolger et al., 2012; Gallo et al., 2022; Mettouris et al., 2016), including those with non-standardized images from Thornicroft's giraffe (*Giraffa camelopardalis thornicrofti*) for instance (Halloran et al., 2015). Halloran et al. (2015) found that background complexity was the only factor that negatively affected matching success, rather than animal orientation. However, as their study was conducted with giraffes, they photographed the animals from the side and not from above like adult European fire salamanders. When European fire salamanders are photographed from above, their bodies may be bended or stretched, as it is the case in our dataset. This is likely to have reduced the performance of Wild-ID.

We found the lowest FRR1 and FRR10 values in ManderMatcher (9.81% and 5.31%, respectively) and the lowest FRR21 value in ARW (3.98%). ARW found 77.3% of recaptures, which is the highest Recognition Rate among the software compared. However, other studies reported Recognition Rates as high as 98%-99% and 100% (using e.g., Wild-ID Mettouris et al.,

2016; or I3S Sacchi et al., 2010). Bendik et al. (2013) found that the quality of the dataset plays an important role as they demonstrated that the FRR for a good quality dataset was 20 times lower than for a poor-quality dataset. However, in our adult dataset the quality differed substantially between the pictures as they were taken under non-standardised conditions in the field, but both software were able to match pictures reliably.

All software tested suggest potential matches and then require confirmation of a match by the users eye. However, even the best performing software, the ARW, was only able to identify 77.3% of all recaptures. For our dataset, which contained an *a priori* unknown number of recaptures, we found that the combination of visual and automated inspection led to the highest Recognition Rate. Visual inspection resulted in an RR of 93.94%, and we defined the combination of visual and computer-aided inspection to be 100% of all recaptures. Visual inspection is by far the most time-consuming method, which can hardly be applied to large datasets. Thus, time efficient and less error-prone automated photographic identification of individuals is an indispensable tool for ecological research and conservation biology while one needs to assume that in non-standardised datasets not 100% of all actual recaptures will be found.

Conclusion

Taking images under standardized conditions plays a major role in the success of recognition software. In contrast to adult images, pictures of larvae will always be taken under more or less standardized conditions, since each larva has to be removed from its water body in order to take a picture of the side of the body as it was the case in our study. This might explain why both ARW and Wild-ID performed better in matching larval than adult images, as the latter were not taken in a standardized way. We assume that (especially in citizen science projects) most pictures of adult European fire salamanders are not taken under standardized conditions, as the animals often move when approached and therefore many images are likely to be taken opportunistically rather than in a controlled manner.

The newly launched ARW software offers several benefits in the picture matching process. It performed very well for the larval dataset and showed the best recapture rate for the adult dataset, thus users only need to use one software to process images of both life stages. Moreover, it is the only software (among the compared) that does not require any image pre-process prior to detect matches. In this way, ARW can be easily used for small and large datasets. Given that it is available online, it also offers the opportunity to involve citizen scientist. In

conclusion, the time efficiency that ARW offers and considering the good performance of the software, it is a helpful tool that can be used for the monitoring of larval and adult European fire salamander populations and thus serve species conservation.

Acknowledgements

We thank Lara Gemeinhardt and the other students that were involved in taking the pictures of adult fire salamanders in the Kruppwald (permission number 59-5-1-53 (1398), Untere Naturschutzbehörde, Essen) as well as the students that helped checking for recaptures by eye and with software.

Author contribution

LS, KP, SS, PO, MV, BAC conceived the study; LS, KP, PO, MV, BAC created the design of the study, KP & CF created the picture datasets, LS & CF performed the picture matching, LS performed the data analysis and wrote the first version of the manuscript. All authors contributed to the final version of the manuscript.

Funding

This research was conducted as part of the CRC TRR 212 (NC[3]) – Project number 316099922 and 396777092 funded by the German Research Foundation (DFG).

Availability of data

Picture datasets used are available from the respective co-authors upon request.

Conflict of interest

The department of Behavioural Ecology from Bielefeld University placed the order for the Amphibian & Reptile Wildbook software together with NC3 (Collaborative research centre "A Novel Synthesis of Individualisation across Behaviour, Ecology and Evolution: Niche Choice, Niche Conformance, Niche Construction (NC3)" founded by the DZG) and Heike Pröhl and Mirjam Nadjafzadeh, who received funding from the Deutsche Gesellschaft für Herpetologie und Terrarienkunde (DGHT). They are not receiving any kind of payment or other benefits in relation with the Amphibian & Reptile Wildbook.

Literature

Aevarsson, U., Graves, A., Carter, K.C., Doherty-Bone, T.M., Kane, D., Servini, F., Tapley, B., Michaels, C.J., 2022. Individual Identification of the Lake Oku Clawed Frog (Xenopus longipes) Using a Photographic Identification Technique. Herpetological Conservation and Biology 17, 67–75.

Arzoumanian, Z., Holmberg, J., Norman, B., 2005. An astronomical pattern-matching algorithm for computer-aided identification of whale sharks Rhincodon typus: Computer-aided identification of whale sharks. Journal of Applied Ecology 42, 999–1011. https://doi.org/10.1111/j.1365-2664.2005.01117.x

Baker, J., Allain, S., 2020. Photographic identification of individual Barred Grass Snakes Natrix helvetica. Transactions of the Norfolk and Norwich Naturalists' Society 53, 4.

Bendik, N.F., Morrison, T.A., Gluesenkamp, A.G., Sanders, M.S., O'Donnell, L.J., 2013. Computer-Assisted Photo Identification Outperforms Visible Implant Elastomers in an Endangered Salamander, Eurycea tonkawae. PLoS ONE 8, e59424. https://doi.org/10.1371/journal.pone.0059424

Bloch, N., Irschick, D.J., 2005. Toe-Clipping Dramatically Reduces Clinging Performance in a Pad-Bearing Lizard (Anolis carolinensis). Journal of Herpetology 39, 288–293. https://doi.org/10.1670/97-04N

Blount, D., Gero, S., Van Oast, J., Parham, J., Kingen, C., Scheiner, B., Stere, T., Fisher, M., Minton, G., Khan, C., Dulau, V., Thompson, J., Moskvyak, O., Berger-Wolf, T., Stewart, C.V., Holmberg, J., Levenson, J.J., 2022. Flukebook: an open-source AI platform for cetacean photo identification. Mamm Biol. https://doi.org/10.1007/s42991-021-00221-3

Bolger, D.T., Morrison, T.A., Vance, B., Lee, D., Farid, H., 2012. A computer-assisted system for photographic mark-recapture analysis: Software for photographic mark-recapture. Methods in Ecology and Evolution 3, 813–822. https://doi.org/10.1111/j.2041-210X.2012.00212.x

Cruickshank, S.S., Schmidt, B.R., 2017. Error rates and variation between observers are reduced with the use of photographic matching software for capture-recapture studies. Amphib Reptilia 38, 315–325. https://doi.org/10.1163/15685381-00003112

Davis, H.-P., VanCompernolle, M., Dickens, J., 2019. Effectiveness and Reliability of Photographic Identification Methods For Identifying Individuals of a Cryptically Patterned Toad. Herpetological Conservation and Biology 9.

Donnelly, M.A., Guyer, C., Juterbock, J.E., Alford, R.A., 1994. Techniques for Marking Amphibians, in: Measuring and Monitoring Biological Diversity: Standard Methods for Amphibians. Smithsonian Institution Press., pp. 277–284.

Drechsler, A., Helling, T., Steinfartz, S., 2015. Genetic fingerprinting proves cross-correlated automatic photo-identification of individuals as highly efficient in large capture-mark-recapture studies. Ecol Evol 5, 141–151. https://doi.org/10.1002/ece3.1340

Drew, M.M., Hartnoll, R.G., Hansson, B.S., 2012. An improved mark-recapture method using passive integrated transponder (PIT) tags in Birgus latro (Linnaeus, 1767) (Decapoda, Anomura). Crustac 85, 89–102. https://doi.org/10.1163/156854012X623656

Eitam, A., Blaustein, L., 2002. Noninvasive individual identification of larval Salamandra using tailfin spot patterns. Amphibia-Reptilia 23, 215–219.

Faul, C., Wagner, N., Veith, M., 2022. Successful automated photographic identification of larvae of the European Fire Salamander, Salamandra salamandra. Salamandra 58, 52–63.

Gallo, J.A., Abba, A.M., Superina, M., 2022. Individual identification of armadillos (Mammalia, Cingulata) using a photo-identification software. Mamm Biol. https://doi.org/10.1007/s42991-022-00260-4

Goedbloed, D., Segev, O., Küpfer, E., Pietzsch, N., Matthé, M., Steinfartz, S., 2017. Evaluation of a new AmphIdent module and sources of automated photo identification errors using data from Salamandra infraimmaculata. Salamandra 53, 314–318.

Gould, J., Taylor, J., Davies, B., Donelly, R., Schmahl, K., Bugir, C.K., Beranek, C.T., McGregor, J., Mahony, S.V., Seeto, R., Upton, R., McHenry, C., Callen, A., 2023. Tadpole fingerprinting: Using tail venation patterns to photo-identify tadpole individuals of a threatened frog. Austral Ecology 48, 585–599. https://doi.org/10.1111/aec.13286

Gould, W.R., Kendall, W.L., 2014. Capture-Recapture Methodology, in: Wiley StatsRef: Statistics Reference Online. pp. 1–13.

Halloran, K.M., Murdoch, J.D., Becker, M.S., 2015. Applying computer-aided photo-identification to messy datasets: a case study of Thornicroft's giraffe (Giraffa camelopardalis thornicrofti). Afr. J. Ecol. 53, 147–155. https://doi.org/10.1111/aje.12145

Heemeyer, J.L., Homyack, J.A., Haas, C.A., 2007. Retention and Readability of Visible Implant Elastomer Marks in Eastern Red-Backed Salamanders (Plethodon cinereus). Herpetological Review 38, 425–428.

IUCN, 2022. The IUCN Red List of Threatened Species. Version 2022-2. URL https://www.iucnredlist.org/

IUCN SSC Amphibian Specialist Group, 2022. Salamandra salamandra. The IUCN Red List of Threatened Species 2022.

Knox, C.D., Cree, A., Seddon, P.J., 2013. Accurate identification of individual geckos (Naultinus gemmeus) through dorsal pattern differentiation. New Zealand Journal of Ecology 37, 60–66.

Lötters, S., Wagner, N., Albaladejo, G., Böning, P., Dalbeck, L., Düssel, H., Feldmeier, S., Guschal, M., Kirst, K., Ohlhoff, D., Preißler, K., Reinhardt, T., Schlüpmann, M., Schulte, U., Schulz, V., Steinfartz, S., Twietmeyer, S., Veith, M., Vences, M., Wegge, J., 2020. The amphibian pathogen Batrachochytrium salamandrivorans in the hotspot of its European invasive range: past – present – future. Salamandra 56, 173–188.

Lowe, D.G., 2004. Distinctive Image Features from Scale-Invariant Keypoints. International Journal of Computer Vision 60, 91–110.

Marshall, A.D., Pierce, S.J., 2012. The use and abuse of photographic identification in sharks and rays. Journal of Fish Biology 80, 1361–1379. https://doi.org/10.1111/j.1095-8649.2012.03244.x

Matthé, M., Sannolo, M., Winiarski, K., Spitzen - van der Sluijs, A., Goedbloed, D., Steinfartz, S., Stachow, U., 2017. Comparison of photo-matching algorithms commonly used for photographic capture-recapture studies. Ecol Evol 7, 5861–5872. https://doi.org/10.1002/ece3.3140

Mettouris, O., Megremis, G., Giokas, S., 2016. A newt does not change its spots: using pattern mapping for the identification of individuals in large populations of newt species. Ecol Res 31, 483–489. https://doi.org/10.1007/s11284-016-1346-y

Oswald, P., Schulte, L., Tunnat, B., Caspers, B.A., 2023. Population monitoring of European fire salamanders (Salamandra salamandra) with a new photo-recognition software. Salamandra 59, 179–197.

Perry, G., Wallace, M.C., Perry, D., Curzer, H., Muhlberger, P., 2011. Toe Clipping of Amphibians and Reptiles: Science, Ethics, and the Law. Journal of Herpetology 45, 547–555. https://doi.org/10.1670/11-037.1

Roberts, L.S., Feuka, A.B., Muths, E., Hardy, B.M., Bailey, L.L., 2021. Trade-offs in initial and long-term handling efficiency of PIT-tag and photographic identification methods. Ecological Indicators 130, 108110. https://doi.org/10.1016/j.ecolind.2021.108110

Rossi de Gasperis, S., Carpaneto, G.M., Nigro, G., Antonini, G., Chiari, S., Cini, A., Mancini, E., Mason, F., Mosconi, F., Redolfi De Zan, L., Roversi, P.F., Sabbatini Peverieri, G., Solano, E., Campanaro, A., 2017. Computer-aided photographic identification of Rosalia alpina (Coleoptera: Cerambycidae) applied to a mark-recapture study. Insect Conserv Divers 10, 54–63. https://doi.org/10.1111/icad.12199

Sacchi, R., Scali, V., Pellitteri-Rosa, D., Pupin, F., Gentilli, A., Tettamanti, S., Cavigioli, L., Racina, L., Maiocchi, V., Galeotti, P., Fasola, M., 2010. Photographic identification in reptiles: a matter of scales. Amphib Reptilia 31, 489–502. https://doi.org/10.1163/017353710X521546

Sandvoß, M., Wagner, N., Lötters, S., Feldmeier, S., Schulz, V., Steinfartz, S., Veith, M., 2020. Spread of the pathogen Batrachochytrium salamandrivorans and large-scale absence of larvae suggest unnoticed declines of the European fire salamander in the southern Eifel Mountains. Salamandra 56, 215–226.

Sannolo, M., Gatti, F., Mangiacotti, M., Scali, S., Sacchi, R., 2016. Photo-identification in amphibian studies: a test of I3S Pattern. Acta Herpetologica 63–68. https://doi.org/10.13128/ACTA_HERPETOL-17198

Schlüpmann, M., Veith, M., 2020. Feuersalamander (Salamandra salamandra), in: Rote-Liste-Gremium Amphibien Und Reptilien (Eds): Rote Liste Und Gesamtartenliste Der Amphibien (Amphibia) Deutschlands. Naturschutz und Biologische Vielfalt, pp. 34–35.

Schulte, U., Küsters, D., Steinfartz, S., 2007. A PIT tag based analysis of annual movement patterns of adult fire salamanders (Salamandra salamandra) in a Middle European habitat. Amphib Reptilia 28, 531–536. https://doi.org/10.1163/156853807782152543

Seber, G.A.F., Schofield, M.R., 2019. Capture-Recapture: Parameter Estimation for Open Animal Populations, Statistics for Biology and Health. Springer International Publishing, Cham. https://doi.org/10.1007/978-3-030-18187-1

Speybroeck, J., Steenhoudt, K., 2017. A pattern-based tool for long-term, large-sample capture-mark-recapture studies of fire salamanders Salamandra species (Amphibia: Urodela: Salamandridae). Acta Herpetologica 55–63. https://doi.org/10.13128/ACTA_HERPETOL-20147

Stuart, S.N., Chanson, J.S., Cox, N.A., Young, B.E., Rodrigues, A.S.L., Fischman, D.L., Waller, R.W., 2004. Status and Trends of Amphibian Declines and Extinctions Worldwide. Science 306, 1783–1786. https://doi.org/10.1126/science.1103538

Thiesmeier, B., 2004. Der Feuersalamander. Laurenti.

Van Tienhoven, A.M., Den Hartog, J.E., Reijns, R.A., Peddemors, V.M., 2007. A computer-aided program for pattern-matching of natural marks on the spotted raggedtooth shark Carcharias taurus: Computer-aided photo-identification of C. taurus. Journal of Applied Ecology 44, 273–280. https://doi.org/10.1111/j.1365-2664.2006.01273.x

Wagner, N., Pfrommer, J., Veith, M., 2020. Comparison of different methods to estimate abundances of larval fire salamanders (Salamandra salamandra) in first-order creeks. Salamandra 56, 265–274.

Figure 1

Click here to access/download;Figure;Fig1.jpg

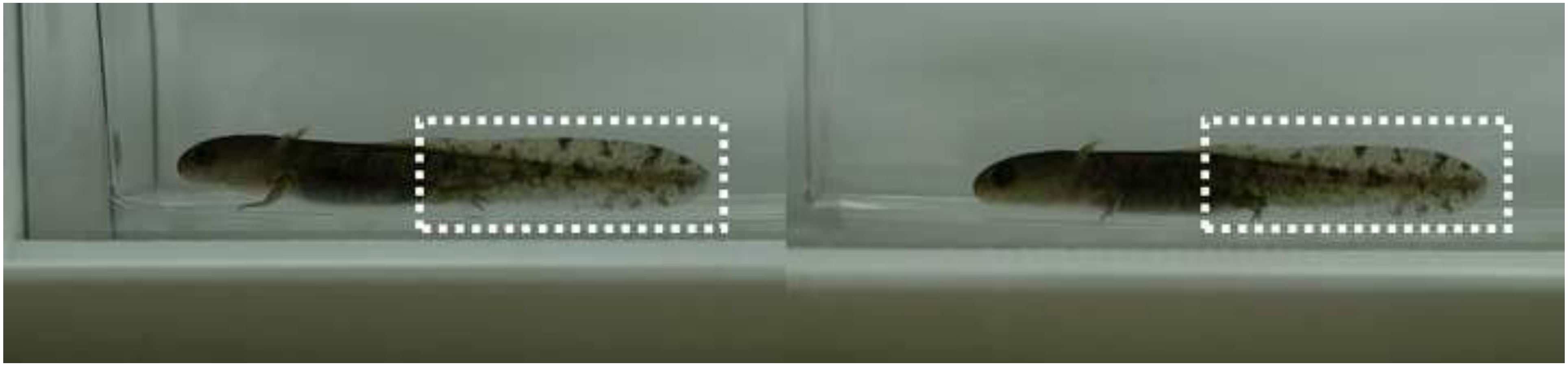

Supplement: Attachment — Submitted filename: PONE-D-23-31936_reviewer_rev.pdf [file pone.0298285.s001.pdf]
