# Supplementary material for: Performance of different automatic photographic identification software for larvae and adults of the European fire salamander
Source: PLoS One. 2024 Apr 4;19(4):e0298285. doi: 10.1371/journal.pone.0298285 (PMC10994360; doi:10.1371/journal.pone.0298285)
Supplement: Attachment — Submitted filename: Response_to_Reviewers.docx [file pone.0298285.s002.docx]

Dear Editor and Reviewers,

Thank you very much for your useful feedback on our manuscript. We appreciate your time and effort to improve this manuscript.

Please find below in red our responses to your comments with the corresponding lines from the updated manuscript (with track changes).

**Reviewer #1**

L26 tautological maybe change with "Alternative low-impact techniques such as..."

We’ve changed it, thanks. L 25 “Alternatives are low-impact techniques such as the use of photos for individual identification, for species with stable distinctive phenotypic traits.”

L27 please rephrase with something more concise, like "...species with stable distinctive phenotypic traits"

We’ve changed it, thanks. Please see previous comment.

L53 I would say are among the most used methods, as recently an even less invasive method demonstrated to be highly reliable to estimate populations size, even without handling individuals: repeated counts. This method is even easier to use than CMR. I would then suggest to highlight more the advantages that CMR can give, such as information on growth rate, home ranges...

Ficetola, G. F., B. Barzaghi, A. Melotto, M. Muraro, E. Lunghi, C. Canedoli, E. Lo Parrino, V. Nanni, I. Silva-Rocha, A. Urso, M. A. Carretero, D. Salvi, S. Scali, G. Scarì, R. Pennati, F. Andreone and R. Manenti (2018). "N-mixture models reliably estimate the abundance of small vertebrates." Scientific Reports 8: 10357.

We’ve rephrased it, thanks. L 51 “Capture-mark-recapture (CMR) studies are among the most used methods to estimate abundance of animals, to track life-histories, give information on growth rate, and movements of individuals and to monitor the dynamic of communities and are a useful tool to estimate population sizes (5–9).”

L79 The Authors should mention also the use of probably the best tagging method, visual implant elastomers, widlely used on frogs and salamanders species, which can last for years and can be also retained after larvae metamorphosis

Grant, E.H.C. (2008). Visual implant elastomer mark retention through metamorphosis in amphibian larvae. Journal of Wildlife Management 72:1247–1252.

Lunghi, E. and G. Bruni (2018). "Long-term reliability of Visual Implant Elastomers in the Italian cave salamander (Hydromantes italicus)." Salamandra 54(4): 283-286.

We’ve added the study, thanks. However, we do not agree with considering it the best tagging method. In Lunghi & Bruni (2018) they could only recover 4 out of 77 tagged salamanders. Indeed, the 4 were not harmed but it remained unknown what happened to the rest so I would argue that it cannot be ruled out that individuals died because of the marking or the wounds that the markings left.

L 71 “Also, Visual Implant Elastomers (VIE) marks were recovered successfully over a period of five years of individually marked Italian cave salamander (Hydromantes italicus)(17).”

L92 It should be mentioned that its efficiency is limited to well-defined patterns. Indeed, as an example there are European cave salamanders: both ventral and dorsal pattern may be used for individual recognition, but only the former can be done using softwares

Lunghi, E., D. Romeo, M. Mulargia, R. Cogoni, R. Manenti, C. Corti, G. F. Ficetola and M. Veith (2019). "On the stability of the dorsal pattern of European cave salamanders (genus Hydromantes)." Herpetozoa 32: 249-253.

Renet, J., L. Leprêtre, J. Champagnon, and P. Lambret. 2019. Monitoring amphibian species with complex chromatophore patterns: a non-invasive approach with an evaluation of software effectiveness and reliability. Herpetological Journal 29:13–22.

We added the first study, thanks. L 82 “However, the specific pattern of a species needs to be tested first, if it is reliable over time to use for individual identification (1).“

Table1 maybe tested on? otherwise it seems that you can employ dataset only of the mentioned species

So far, only the mentioned species are available for the named software programmes so we guess “availability” is a proper term here. The ARW for example can right now only be used for three species. It can be expanded to other species but that would require programming and training the software first.

**Reviewer #2**

Line 26: There is a mismatch in singular vs. plural here. I would recommend changing it to “An alternative can be found among non-invasive techniques…” or something like that.

We’ve already changed the sentence according to the recommendation of the other reviewer. L 25 “Alternatives are low-impact techniques such as the use of photos for individual identification, for species with stable distinctive phenotypic traits.”

Line 32: I think that “computer-based” is redundant and unnecessary here.

We’ve removed it, thanks.

Lines 34–35: As I understand it, this is a current limitation of this software. But it may not be permanent. So I would say, “…larvae can currently only be identified…”. Perhaps new modules will become available in the future, so to make sure this study remains accurate through time, I would encourage the authors to rephrase this.

We’ve changed it, thanks. L 33 “While adults can be identified by all five software, European fire salamander larvae can currently only be identified by two of the five (ARW and Wild-ID).”

Line 36: Change “larvae” to “larval”. In general, this manuscript is well-written and the English is clear. There are several places where it could use some minor improvement, but I do not view this as a barrier to publication. If the authors are interested, the Society for the Study of Amphibians and Reptiles does offer a Manuscript Review Service for this purpose (see details here: https://ssarherps.org/publications/manuscript-review-service/).

We’ve changed it, thanks.

Line 51: There is an updated analysis that was recently published on this topic. “Ongoing declines for the world’s amphibians in the face of emerging threats” by Luedtke et al. (2023). The authors may wish to cite this for the latest information.

We’ve added this study, thanks. L 49 “Worldwide, 41% of the amphibian species suffer from population declines, and the conservation status of an additional 11% is not even known due to deficient data (1–3).”

Line 61: Unless I am misunderstanding, I think these are more often referred to as “bands” than “rings”.

In both north and south America, it is indeed referred to as “bands”, whereas here in Europe it is called bird “ringing”. As the fire salamander is distributed here, we would like to stick with the “European” term.

Lines 119–120: It is not technically wrong to say “…the following five software”, but it would be more common in English to say “…the following five software programs”, even if it is unnecessarily redundant in meaning. Just an idiosyncrasy of the language. I don’t think a change here (and in other places throughout the manuscript where this comment is relevant) is necessary, but the authors may want to consider it.

We’ve added it, thanks. L 108 “We used the following five software programmes”

Lines 182–184: I think the authors should consider citing “Computer-assisted photo identification outperforms visible implant elastomers in an endangered salamander, Eurycea tonkawae” by Bendik et al. (2013) as another example of Wild-ID being applied to salamanders. Perhaps the information in the discussion of this paper would also prove valuable.

We’ve added the study, thanks. L 163 “Wild-ID has been successfully used to study amphibians before, e.g., the Yellow-bellied toad, the Jollyville Plateau salamander (Eurycea tonkawae) and the Lake Oku clawed frog (Xenopus longipes) (12,19,27).”

Line 186: As the authors describe earlier, image processing is recommended for Wild-ID, but it is not required. This should be clearer in the table.

We have changed it, thanks. Table 1:

|  | Image processing required? | Available for which species? |
| --- | --- | --- |
| Amphibian & Reptile Wildbook (ARW) | No | Yellow-bellied toad (*Bombina variegata*), European fire salamander (*Salamandra salamandra*), Near-eastern fire salamander (*S. infraimmaculata*) |
| AmphIdent | Yes | Great crested newt (*Triturus cristatus*), European fire-bellied toad (*B. bombina*), the Yellow-bellied toad, European fire salamander, the Marbled salamander (*Ambystoma opacum*) |
| I3S pattern+ | Yes | Every species with individual pattern |
| ManderMatcher | Yes | Fire salamander species |
| Wild-ID | Recommended | Every species with individual pattern |

Line 231: Recognition Rate should also be defined clearly here. The results are hard to interpret otherwise.

We added a definition, thanks. L 213 “We defined the Recognition Rate as the proportion of recaptures from the full dataset that was successfully identified as a match by the software.“

Line 233: Does “we used the existing FRR from Faul et al. (2022)” mean that the authors simply adopted the definitions from this paper, but recalculate these values? Or that they simply report the exact same values originally calculated in that study?

Here we report the same values from Faul et al 2022 with Wild-ID. We also used the same dataset for the further testing in the Amphibian and Reptile Wildbook. L 214 “For the larval dataset in Wild-ID, we report the existing FRR from Faul et al. (2022).”

Line 256: Should this say “FRR” instead of “FFR”? Likewise on Lines 257, 267, and several other places.

Thanks for pointing this out. That was indeed a typing error that occurred several times. We have changed it in the whole document.

Line 296: As noted earlier, I think it is critical that “Recognition Rate” (RR) is defined. I would have assumed that it means something like “the probability of identifying a true match somewhere in the list of potential matches”. However, this doesn’t appear to be possible. For example, an FRR1 of 15.12 in Wild-ID would suggest that the correct match *was* identified in the first position for ~85% of photographs. However, the RR is only 37.88. This seems to mean that my intuition is wrong about the definition of RR. I think that a better explanation of this should be a priority.

Please see comment above.

Line 337: I think the authors may consider expanding their discussion in two ways: 1) Would there be value in combining several of these methods? The authors acknowledge that manual visual inspection + software is very effective. But what about, for example, ARW + Wild-ID? Would that improve the ability to do this reliably?; and 2) This seems like a field that is likely to change quickly and dramatically with the advent of modern artificial intelligence tools. Is there reason to believe that all of these existing tools (i.e., the ones compared in this study) might be replaced in the near future? I am not asking the authors to speculate wildly, but a brief discussion of the future directions for these methods might prove useful.

We’ve added two sentences here. L 306 “Another possibility to improve reliability of the software could be to combine results from two software programmes, like Wild-ID and ARW for instance. However, different software have different requirements for the image processing and using more than one software can also be considered quite time consuming and thus not very effective.”

We’ve also added one sentence in the conclusion. We believe that the ARW will also in the future be a valuable tool as the algorithm that is used here keeps learning and getting better the more it is used. L 327 “In addition, it is likely to deliver even better results in the future as the hot spotter algorithm gets better (“learns”) with more images being matched.”

Line 374: This does not appear to satisfy the PLOS Data policy. I recommend making these underlying data available in a public repository.

Please see new data availability statement L 346 “The larval dataset is available here <https://doi.org/10.6084/m9.figshare.24999353.v1>, and the adult dataset is available here <https://doi.org/10.6084/m9.figshare.24998690.v1>.”
